# Establishment of lateral organ asymmetries in the invertebrate chordate, *Ciona intestinalis*

**DOI:** 10.1186/s13227-017-0075-9

**Published:** 2017-07-25

**Authors:** Karl Palmquist, Brad Davidson

**Affiliations:** Department of Biology, Swarthmore College, 500 College Ave., Swarthmore, PA 19081 USA

**Keywords:** Left/right patterning, Chordate evolution, Tunicates, Heart development, Endoderm development, Nodal signaling

## Abstract

**Background:**

The evolutionary emergence and diversification of the chordates appear to involve dramatic changes in organ morphogenesis along the left/right axis. However, the ancestral chordate mechanism for establishing lateral asymmetry remains ambiguous. Additionally, links between the initial establishment of lateral asymmetry and subsequent asymmetries in organ morphogenesis are poorly characterized.

**Results:**

To explore asymmetric organ morphogenesis during chordate evolution, we have begun to characterize left/right patterning of the heart and endodermal organs in an invertebrate chordate, *Ciona intestinalis*. Here, we show that *Ciona* has a laterally asymmetric, right-sided heart. Our data indicate that cardiac lateral asymmetry requires H^+^/K^+^ ion flux, but is independent of Nodal signaling. Our pharmacological inhibitor studies show that ion flux is required for polarization of epidermal cilia and neurula rotation and suggest that ion flux functions synergistically with chorion contact to drive cardiac laterality. Live imaging analysis revealed that larval heart progenitor cells undergo a lateral shift without displaying any migratory behaviors. Furthermore, we find that this passive shift corresponds with the emergence of lateral asymmetry in the endoderm, which is also ion flux dependent.

**Conclusions:**

Our data suggest that ion flux promotes laterally asymmetric morphogenesis of the larval endoderm rudiment leading to a passive, Nodal-independent shift in the position of associated heart progenitor cells. These findings help to refine hypotheses regarding ancestral chordate left/right patterning mechanisms and how they have diverged within invertebrate and vertebrate chordate lineages.

**Electronic supplementary material:**

The online version of this article (doi:10.1186/s13227-017-0075-9) contains supplementary material, which is available to authorized users.

## Background

The emergence of diverse bilaterian body plans is thought to reflect variable deployment of developmental regulatory modules along ancestral signaling axes. Widely divergent bilaterian clades appear to deploy the same set of signaling pathways to coordinate embryonic growth along a cartesian axial system. The anterior/posterior axis is dictated by Wnt signaling, dorsal/ventral by BMP signaling, and left/right (LR) by Nodal signaling. Broad conservation of axial signaling is thought to reflect a deeply homologous, ancestral embryonic patterning mechanism at the base of the bilateria. However, the process by which the initial axial signals coordinate subsequent organ morphogenesis and how this process has been altered to generate novel traits remains poorly defined. This is particularly true for the more recently characterized LR molecular axis [1]. Insights into LR axis specification are essential for deciphering patterns of evolutionary diversification. Additionally, errors in embryonic LR axis formation underlie prevalent congenital abnormalities in organ form and function [2, 3].

Left–right patterning is a complex, multi-step process [4]. First, lateral symmetry must be broken. This initial asymmetry then generates a stable lateralized signaling gradient that is subsequently relayed to adjacent tissues. Finally, lateral differences in exposure to axial signals drive differential programs of organ morphogenesis. Evolutionary conservation of this complex pathway is stage specific with an hourglass-like conformation, as stringent conservation appears to occur primarily in the central Nodal signaling step. At the bottom of the hourglass, there is enormous variability of morphogenetic programs directed by axial signals. At the top of the hourglass, variability in the initial symmetry-breaking mechanisms, even within the vertebrates, has derailed attempts to reconstruct ancestral LR patterning mechanisms. In *Xenopus,* zebrafish and mouse, symmetry is broken by ciliary flow [5–7]. Polarized cilia drive asymmetric flow across a midline LR organizer tissue, termed the gastrocoel roof plate in *Xenopus* [7], the node in mice [5, 8] or Kupffer’s vesicle in Zebrafish [6]. Flow across the LR organizer generates localized signaling gradients including fibroblast growth factor (FGF), Sonic hedgehog (SHH), Nodal and Ca^2+^ ions [9–13]. Through a poorly characterized relay mechanism, this midline axial signal drives asymmetric Nodal signaling in the adjacent lateral plate mesoderm. In contrast, the initial symmetry disruption in chick and pig embryos appears to involve directed rotation of organizer tissue instead of ciliary flow [14]. Intriguingly, establishment of the initial LR asymmetry in both frog and chick embryos requires ion flux driven by a H^+^/K^+^-ATPase [14, 15]. The role of ion flux in lateral patterning remains controversial. The initial work in *Xenopus* embryos indicated that asymmetric localization of the ATPase during early cleavage stages led to an early break in symmetry [15]. Recent studies contradict these results, suggesting that ion flux contributes to LR patterning by participating in node ciliogenesis [16]. H^+^/K^+^-ATPase dependent ion flux is also required for establishing lateral asymmetry in tunicates and sea urchins [17, 18]. Studies in sea urchins and tunicates suggest that ion flux functions upstream of Nodal signaling during lateral patterning [17, 18]. However, the specific role of ion flux in tunicate lateral organ morphogenesis has not been investigated.

Although the initial symmetry-breaking mechanisms vary, Nodal signaling is thought to play a highly conserved role in bilaterian left/right patterning preserved through 500 million years of evolutionary diversification. The initial studies of vertebrate LR patterning revealed a key role for Nodal and downstream expression of the transcription factor Pitx [12, 19–22]. Subsequent studies revealed that Nodal/Pitx also regulates LR patterning in the invertebrate chordates, including both amphioxus and tunicates [23, 24] and more broadly within non-chordate deuterostomes (sea urchins) [18] and protostomes (molluscs) [25]. Most recently, a Nodal-related gene was shown to regulate *pitx* expression and lateral branching in hydra [26], suggesting that Nodal-dependent lateral patterning represents an ancestral eumetazoan character state. In contrast, genes encoding Nodal are not represented in the ecdysozoan taxa [1]. There is also some evidence for alternative Nodal-independent mechanisms underlying LR patterning [27]. Despite these inconsistencies, Nodal’s status as the linchpin of LR patterning has remained largely unchallenged and research has predominantly focused on deciphering upstream and downstream mechanisms.

Despite an intensive and productive research effort, numerous aspects of vertebrate left/right patterning remain poorly described. Crucial gaps include (1) the precise contributions of ciliary flow, mechanosensory cilium, Ca^2+^ ion flux and midline FGF, SHH and Nodal morphogen gradients in establishing an initial, robust signaling axis, (2) the relay system by which the initial midline gradient propagates to lateral portions of the embryo, (3) the cascading series of downstream effects that lateralize organ morphogenesis producing striking asymmetries in heart and gut formation. One highly productive line of inquiry regarding lateralized organogenesis focuses on zebrafish heart formation. As in all vertebrates, the zebrafish heart primordium begins as a midline structure. As the heart tube begins to form, it tilts to the left. Subsequently, the zebrafish heart undergoes lateralized looping, similar to that in other vertebrate embryos [28]. A series of studies has revealed the precise means by which Nodal signaling in the left lateral plate participates in a regulatory circuit with BMP and Nodal to drive tilting of the heart primordium [29, 30]. Intriguingly, subsequent looping morphogenesis has been revealed to be Nodal independent [27]. This raises the question of what alternative, parallel pathways may function in conjunction with Nodal to drive the full suite of lateralized morphogenetic cell behaviors. To further our understanding of vertebrate organ morphogenesis and the role of LR patterning in chordate evolution, we have initiated a study of LR heart patterning in an invertebrate chordate phylum, the tunicates.

Tunicates are the closest sister group to the vertebrates, making them a key taxon for studying chordate development and evolution. The best studied model of tunicate development is *Ciona intestinalis*. *Ciona* development shares fundamental similarities to that of vertebrate embryos, but within a much simpler cellular and genetic context, enabling clarification of complex shared processes [31]. In particular, the gene regulatory networks and morphogenetic cell behaviors underlying formation of the notochord, neural tube and cardiopharyngeal mesoderm share extensive similarities with orthologous processes in vertebrate embryos [32–37]. Recent studies have begun to produce a high-resolution map of the *Ciona* cardiopharyngeal gene regulatory network and delineate how this network regulates lineage specification and associated cell behaviors underlying early steps of heart development [38–42]. However, the differentiation of *Ciona* cardiomyocytes and subsequent morphogenesis of a functioning organ occurs after metamorphosis and remains poorly characterized. Additionally, early lineage specification and axis formation in *Ciona* have diverged from other chordates, relying heavily on maternal determinants associated with rapid embryogenesis. One consequence of this more “mosaic” developmental mode is a reduced role for key embryonic signaling pathways such as Nodal and BMP in early patterning. Thus, while Nodal signaling has a key organizer function in both vertebrate and amphioxus embryos, contributing to all three embryonic axes, *Ciona* Nodal only participates in early LR patterning [43].

Recent research has begun to elucidate the molecular mechanisms underlying left/right patterning in tunicates. The initial symmetry breakage appears to involve lateral rotation of the neurula stage embryos driven by ectodermal cilia and subsequent lateralized contact with the surrounding chorion [44, 45]. Dechorionation disrupts this process, revealing that chorion contact is required for laterally asymmetric expression of *nodal* in the ectoderm and downstream expression of *pitx*. Disruption of Nodal signaling has been shown to randomize markers of tunicate embryonic lateral asymmetry including directional tail bending and lateralized positioning of a light sensing organ, the ocellus [44]. Disruption of H^+^/K^+^-ATPase function also leads to loss of asymmetric *pitx* expression in *Ciona* [17]. The impact of these manipulations on post-larval organ asymmetries, including the heart and gut, has not been investigated. Indeed, although tunicate organ asymmetries have been repeatedly noted in the literature, whether these asymmetries represent robust directed lateralities has not been investigated.

To unlock the potential use of *Ciona* to investigate laterally asymmetric organ morphogenesis during chordate evolution, we have begun to characterize left/right patterning of the heart and endodermal organs. Here, we show that *Ciona* has a laterally asymmetric, right-sided heart. Our data indicate that this lateral asymmetry requires H^+^/K^+^ ion flux and is independent of Nodal signaling. We propose that ion flux-dependent cilia polarization leads to laterally asymmetric endoderm morphogenesis that drives a passive shift in heart progenitor position.

## Methods

### Embryological techniques

*Ciona* (*Ciona robusta/Ciona intestinalis* type a) [46] adults were collected by M-Rep (San Diego County) and kept at 18 °C in artificial seawater (Crystal Sea Marine Mix) under constant light. Fertilization, dechorionation and electroporation were performed as previously described [47]. All electroporations were performed using 100 µg of each DNA construct to ensure high penetrance. Rearing of juveniles was performed by settling hatching larva (St. 29) on non-coated dishes and allowing development until 4 days post-fertilization (d.p.f.) at 18 °C.

### Inhibitor treatments

Omeprazole (PHR1059, Sigma, 10 mM stock) and SB431542 (S4317, Sigma, 30 mM stock) inhibitor stock dilutions were made using DMSO (D2650, Sigma). All the final dilutions were made by introducing the appropriate volume of stock inhibitor to 10 mL of filtered seawater (FSW) containing embryos. For chorionated embryos, a “spin-down” approach was used to wash off omeprazole. Treated FSW and embryos were placed in 10-mL centrifuge tubes and spun on hand centrifuge 40 times, rinsed, centrifuged 40 times again and rinsed once more in FSW. Dechorionated embryos were rinsed using a transfer pipette three times into fresh dishes with 10 mL FSW.

### Staining and confocal microscopy

Staining for larva and juveniles was performed using an overnight fixation in 0.4% paraformaldehyde in FSW (16% stock; Electron Microscopy Sciences). For phalloidin staining, larva and juveniles were incubated for 5 min in 0.1% Triton X-100 (Sigma) in FSW. Samples were then washed in 1× PBS + 1% BSA (PBS-B) and 1× PBS + 0.1% Triton (PBTr), followed by two washes in PBS-B. Samples were then incubated on a nutator overnight at 4 °C in 250 µL PBS-B + 1:125 Alexa Fluor 635 phalloidin (Invitrogen). This incubation was followed by two rinses in 1× PBS-B, and samples were mounted in glycerol using a raised coverslip.

Staining for cilia was performed using overnight methanol fixation at −20 °C. Embryos were then rinsed in MeOH/1× PBS + 0.1% Tween-20 (PBTw). Samples were then rinsed 5× in PBTw, followed by a 1-h incubation in PBS-B at room temperature. Samples are then incubated overnight on a nutator at 4 °C in PBS-B + 1:1000 anti-gamma-tubulin (T5326, Sigma) and 1:1000 anti-acetylated tubulin (T7451, Sigma). Samples are then washed 3× in PBTw and then blocked using 2% normal donkey serum (5% v/v; Jackson ImmunoResearch 017-000-121) for 1 h. Samples are then nutated in 1× PBS + 2%NDS + 1:1000 donkey anti-mouse 488 (Invitrogen) and 1:1000 donkey anti-mouse 633 (Invitrogen) overnight at 4 °C. Embryos were then washed 3× in 1× PBS-B and mounted in glycerol. Z-stack images (1-µm sections for juveniles and 0.5-µm sections for larva and embryos) were acquired using a Leica SP5 confocal microscope.

### Larval heart positioning assay

Fixed larva were mounted in 75% glycerol in between a large and small coverslip and then rotated by moving the top coverslip so the ventral surface was facing the inverted microscope objective. Anterior was demarcated by pigment spots and the midline by the larval endostyle rudiment. In all larvae sampled, the middle of the endostyle rudiment was positioned equidistant from both the left and right sides of the larval head, and the line bisecting the larvae from the middle of the endostyle to the middle of the tail demarcated the midline. Using ImageJ (NIH), the distance in micrometer from the middle of the heart cells to the nearest side of the head was measured. We also measured the distance from the middle of the larval head to side of the head containing the heart progenitors. The latter distance was divided by the former distance to provide a cell shift ratio (Fig. 2), and heart cell positioning was categorized based on this ratio.

### Quantification of cilia positioning and length

For quantification of cilia length, images were processed in ImageJ. Cilia were measured from the base of the cilium to the tip, to the nearest hundredth of a micrometer.

For quantification of cilia polarization, cells were measured from the anterior-most point to the posterior-most point. Then, the distance from a cilium’s base to the posterior-most point was measured. If the distance from a cilium’s base to the posterior-most point was either equal to or less than a third the distance from the cell’s anterior-most point to posterior-most point, these cilia were classified as posteriorly polarized.

### Statistical analysis

All analyses were performed in Excel. All *n* values are reported in the text. *P* values are reported in the figure legends for *χ* ^2^ analysis, Wilcoxon rank sum test and student’s *t* test (two sample, unpaired, unequal variance).

## Results

### Directed lateral asymmetry of the *Ciona* heart

**Fig. 1:**
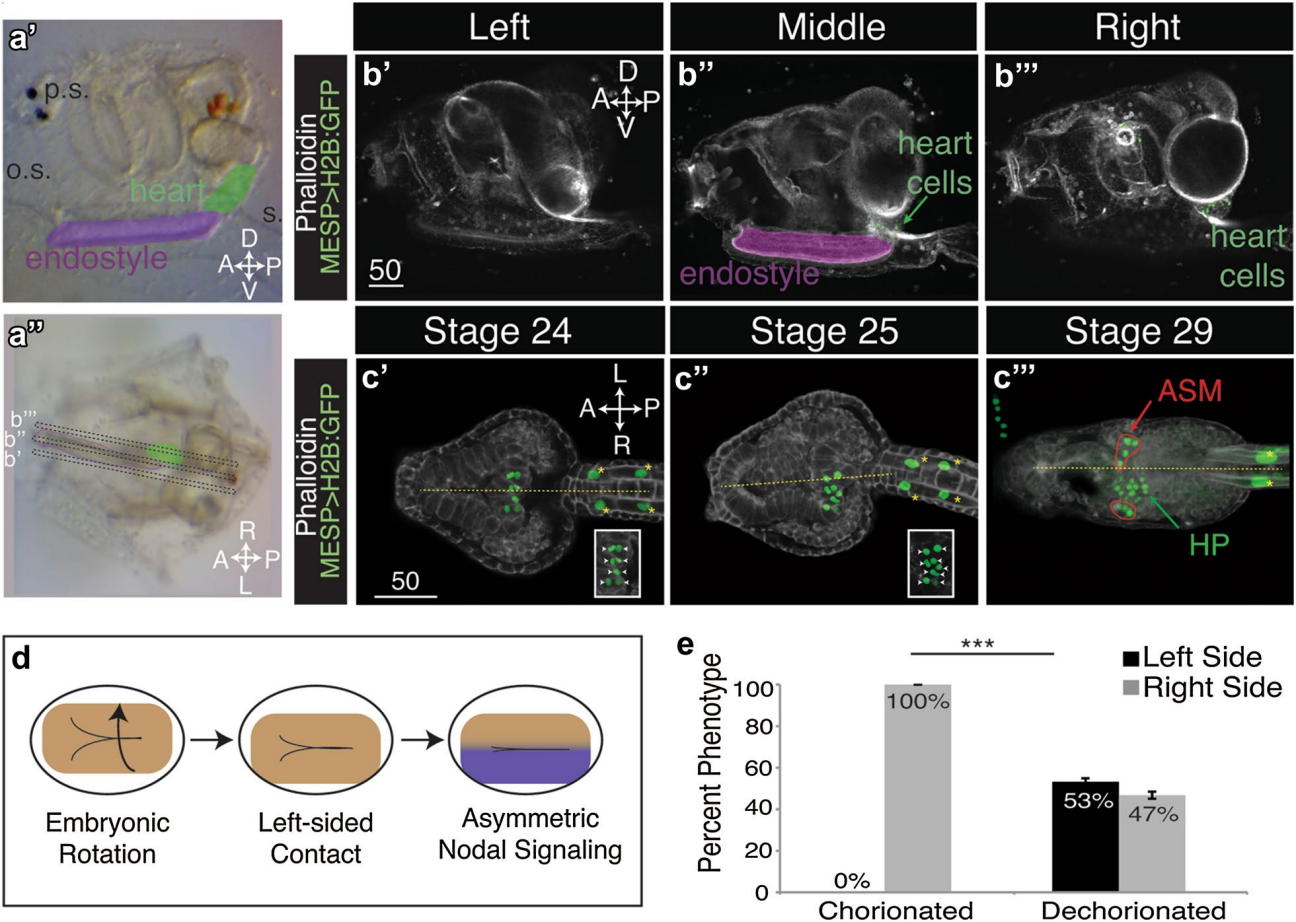
Right-sided heart positioning in *Ciona*. **a′**–**a″** Lateral (**a′**) and dorsal (**a″**) views of a typical *Ciona* juvenile, (o.s.) oral siphon, (p.s.) pigment spots, (s.) stalk. Heart and endostyle are *false-colored*, **(b′**–**c**‴**)** lateral confocal sections of *Ciona* juveniles (**b′**–**b**‴) and ventral projections of late embryonic and larval stages (**c′**–**c**‴). Cell outlines are visualized using phalloidin, and heart founder cell nuclei are transgenically labeled by Mesp > H2B:GFP (*green*). Sections in (**b′**–**b**‴) correspond to lines in (**a″**). Lateral differences in GFP intensity (most evident in **c″**) reflect uneven incorporation of transgenic constructs on the right versus left sides of the embryo. In (**c′**–**c″**), heart progenitor nuclei are shown at high gain in the bottom right and demarcated by *white arrowheads*. Atrial siphon muscle precursors (ASM) are *circled* in *red*, and anterior tail muscle (ATM) cells are marked using *yellow asterisks*. **d** Schematic of neurula rotation and hypothesized mechanism for establishing embryonic left–right axis, and dorsal views are shown. **e** Impact of dechorionation on juvenile heart positioning, and data represent three trials including 71 chorionated samples and 71 dechorionated samples. *P* value (1.4E−12) between chorionated and dechorionated samples was derived using a *χ* ^2^ test. In all images and schematics, anterior is to the *left.* All *scale bars* are in micrometers

The recent characterization of *Ciona* embryonic left–right patterning has failed to ascertain if embryonic asymmetry translates into post-metamorphic asymmetry. Therefore, we examined heart position in *Ciona* juveniles. We assessed juvenile heart laterality using four morphological markers: the endostyle, pigments spots, oral siphon and stalk-like ampulla (Fig. 1a). The endostyle and pigment spots demarcate the ventral and dorsal midlines, respectively, while the oral siphon and ampulla demarcate the anterior–posterior axis. Using these landmarks, we identified the heart as consistently right-sided in wild-type juveniles (Fig. 1a″, e).

We next asked when heart field asymmetry first arises. In tailbud stage embryos, the heart precursors consist of bilateral clusters of heart progenitors that initially fuse symmetrically at the ventral midline [41]. However, heart progenitor positioning along the left–right axis during late tailbud and early larval stages has not been characterized. To fill this gap, we transgenically labeled heart progenitor cell nuclei using the Mesp > H2B:GFP reporter construct (the *Ciona Mesp* enhancer driving expression of a Histone 2B-GFP fusion protein) [33] and examined heart cell position in three stages: late tailbud II (St. 24, 13.5 HPF), late tailbud III (St. 25, 15.9 HPF) and late swimming larva (St. 29, 24 HPF) [48]. At St. 24, the head trunk region is roughly symmetric and the heart progenitor cells are symmetrically distributed along either side of the midline endostyle (Fig. 1c′). By St. 25, the trunk region displays laterally asymmetric lobes (Fig. 1c″). This overall morphological asymmetry is associated with a lateral shift of the heart progenitor cells to one side of the endostyle (Fig. 1c″). During St. 29, the head elongates and the papillae mature, priming larvae for settlement. The heart progenitor pool has divided to produce a medial group of heart precursor cells and is flanked by two lateral clusters of atrial siphon muscle (ASM) precursors (Fig. 1c‴). In these late larvae, cardiac laterality is more evident as the heart precursors have shifted completely off the endostyle-demarcated midline (Fig. 1c‴).

### Dechorionation disrupts lateral heart directionality

We next asked whether chorion contact contributes to heart lateral asymmetry. Studies in the tunicate *Halocynthia roretzi* have shown that removal of the chorion disrupts embryonic left/right patterning leading to midline positioning of the tail and neural complex [44]. Further, studies in *Halocynthia* revealed that lateralized contact with the chorion is required for lateralized expression of *nodal* and subsequent Nodal-dependent embryonic LR patterning (Fig. 1d). In *Ciona,* dechorionation disrupts asymmetric gene expression, leading to bilateral *nodal* expression [17]. However, the impact of dechorionation on embryonic asymmetric morphogenesis has not been characterized. To determine if a chorion-dependent mechanism regulates embryonic LR asymmetry, we examined tail bending in chorionated and dechorionated embryos (St. 23) [48]. In chorionated controls, the tip of the tail was located to the right of the head in all embryos scored (50/50; two trials). When the chorion was removed, nearly half the embryos scored had a reversal in the direction of tail bending (29/60 left; 31/60 right; two trials). Thus, in accordance with data in *Halocynthia* [44], a chorion-dependent mechanism regulates *Ciona* lateral tail directionality (i.e., left- vs. right-sided bending). However, dechorionated *Halocynthia* embryos developed with straight tails [44] while dechorionated *Ciona* embryos never exhibited this laterally symmetric phenotype. The lack of midline tails in dechorionated *Ciona* embryos suggests that tail growth asymmetry is chorion independent. Alternatively, a physical barrier may block midline tail growth.

To determine whether *Ciona* heart laterality is influenced by a chorion-dependent mechanism, dechorionated zygotes were reared through metamorphosis and scored for heart positioning in 4-day-old juveniles. Strikingly, nearly half the juveniles that developed from dechorionated eggs had a left-sided heart (Fig. 1e). These results suggest that the chorion is necessary for lateral heart directionality. However, no midline hearts were observed, indicating that a chorion-dependent mechanism does not contribute to asymmetric positioning. Alternatively, a physical barrier, such as the endostyle, may obstruct midline heart formation.

### Disruption of Nodal signaling does not perturb asymmetric heart positioning

In both *Halocynthia* and *Ciona*, removal of the chorion disrupts the embryonic left/right Nodal signaling axis [17, 44]. We therefore hypothesized that Nodal signaling regulates lateral heart asymmetry downstream of chorion contact. To test this hypothesis, we disrupted Nodal signaling by treating neurula stage embryos (St. 14) [48] with the well-established pharmacological inhibitor SB431542 [24, 27, 49] and examined the impact on both embryonic morphology (tail bending) and heart asymmetry. DMSO controls displayed normal right-sided tail bending (60/60; two trials). However, in embryos treated with 0.050 µM SB431542 we observed randomized tail bending (37/70 left; 33/70 right; two trials). Intriguingly, this treatment did not impact juvenile heart asymmetry. In both DMSO controls and 0.050 µM SB431542-treated samples, the heart consistently formed on the right side (50/50, DMSO; 70/70, SB431542; two trials).

**Fig. 2:**
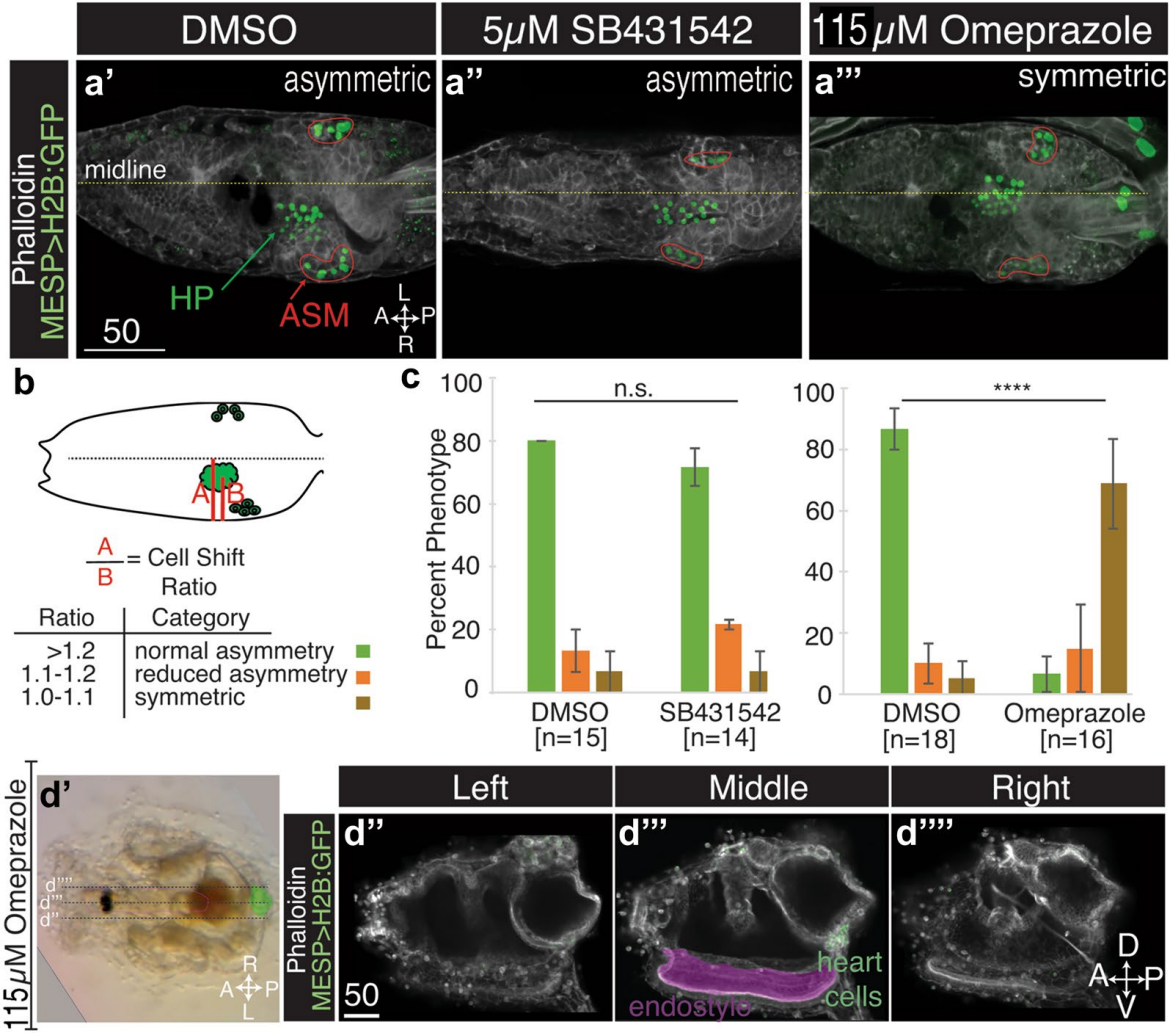
Ion flux is required for laterally asymmetric heart positioning. **a′**–**a**‴ Ventral projections of transgenic Stage 31 larva, and heart founder lineage cells are demarcated by Mesp > H2B:GFP (*green*), including heart precursors (HP) and atrial siphon muscle precursors (ASM). **a′**–**a**‴ Typical asymmetric heart precursor positioning in DMSO controls (**a′**), larva treated with 5 µM SB431542 (**a″**) or larva treated with 115 µM omeprazole (**a**‴). **b** Schematic illustrating cell shift ratio used for scoring heart precursor positioning, as detailed in the “Methods” section. **c** Graphical representation of larval heart position data. *Error bars* represent SEM, and *n* represents total number of larva scored over three-independent trials. Chi-square test derived *P* values comparing control and omeprazole-treated larva (*p* = 2.9E−5) or SB431542-treated samples (*p* = 0.84). **d′**–**d**′′′′ Dorsal view (**d′**) and lateral sections (**d″**–**d**′′′′) of *Ciona* juveniles displaying a midline heart phenotype following omeprazole treatment. In (**d′**), the heart is *false-colored green* and the endostyle is false-colored purple. In lateral sections (**d″**–**d**′′′′), heart cell nuclei are labeled by Mesp > H2B:GFP. In all images, anterior is to the *left*. All *scale bars* are in micrometers

To more strongly inhibit Nodal signaling, we treated *Ciona* neurula stage embryos with 5 µM SB431542. In previous studies, this dosage disrupted left/right asymmetry in *Ciona* and other invertebrate embryos [25, 50, 51] and we confirmed that this treatment abrogated Nodal-dependent lateral *pitx* expression (Additional file 1: Figure S1). In accordance with previous reports [50], we found that this treatment also led to loss of pigment spot asymmetry in the larval cerebral vesicle (data not shown). However, at this dosage embryos were unable to complete metamorphosis and juvenile heart positioning could not be assessed. We therefore examined the position of transgenically labeled heart progenitors in larval stages. To label the heart progenitors, zygotes were dechorionated and electroporated with Mesp > H2B:GFP [33]. Because dechorionation randomizes lateral heart directionality, we could not use this assay to characterize the role of Nodal signaling in *Ciona* lateral heart field directionality. However, we were still able to assess whether Nodal signaling was required for heart field asymmetry. Transgenic Mesp > H2B:GFP larvae were fixed at St. 29 and stained with phalloidin to visualize epidermal structures (Fig. 2a). We quantitatively assessed the degree to which heart progenitors were asymmetrically positioned by generating a displacement ratio for each sample (Fig. 2b). Based on ratios observed in wild-type larvae, larvae displaying heart displacement ratios of 1.2 or greater were classified as normal. Those with ratios of 1.1–1.2 were classified as having reduced asymmetric positioning, and those with ratios less than 1.1 were classified as having symmetric positioning. In accordance with our data from lower doses of SB431542, we found no discernible difference between 5 µM SB431542-treated larva and DMSO controls (Fig. 2a″, c). Together, these data suggest that *Ciona* lateral heart asymmetry is regulated through a chorion and Nodal-independent pathway.

### Neurula stage H^+^/K^+^-ATPase function is required for asymmetric heart positioning and directionality

The apparent Nodal independence of heart lateral asymmetry led us to search for alternative mechanisms. H^+^/K^+^-ATPase-mediated ion flux contributes to left/right patterning in a variety of phyla, including the tunicates [15–17, 20]. We therefore investigated whether H^+^/K^+^-ATPase function contributes to *Ciona* heart lateral asymmetry. Chorionated embryos were treated with the H^+^/K^+^-ATPase inhibitor omeprazole at the early neurula stage (St. 14) and then rinsed at late tailbud stages (St. 23). We first examined the impact of omeprazole treatment on tail bending. In contrast to DMSO controls (53/53, right, three trials), omeprazole treatment randomized tail bending (43/84, left; 41/84, right, three trials). This result aligns with previous data, indicating that ion flux regulates *Ciona* embryonic LR patterning [17].

We next examined the role of H^+^/K^+^-ATPase in lateral heart positioning. Early neurula stage embryos (St. 14) were treated with omeprazole and reared through metamorphosis. In omeprazole-treated juveniles, heart position was randomized (61/116 right, 38/116 left, three trials). Strikingly, we also observed that ~15% of treated embryos developed into juveniles with medial hearts (17/116, Fig. 2d′–d′′′′). We also examined the impact of omeprazole treatment on larval heart precursor positioning. We found that omeprazole treatment had a dramatic, significant impact on heart precursor laterality with >60% of treated larvae displaying symmetric positioning of the heart precursor field (Fig. 2a‴, c). Taken together, these data suggest that ion flux regulates both lateral heart asymmetry and directionality.

Next, we investigated when ion flux is required for lateral heart asymmetry. Studies in *Xenopus* suggest that ion flux may contribute to left/right patterning as early as the four-cell stage [15]. In *Ciona,* a recent report indicates that neurula stage disruption of ion flux results in a dramatic and significant disruption of asymmetric *pitx* expression. These authors also showed that disruption of ion flux from fertilization until the end of gastrulation had a mild, but significant influence on asymmetric *pitx* expression [17]. To examine if there is an earlier role for ion flux in lateral heart asymmetry, we treated newly fertilized *Ciona* zygotes with omeprazole and rinsed off the inhibitor at the end of gastrulation (St. 13). This early omeprazole treatment had no impact on heart positioning (50/50, right, two trials). We also compared heart positioning in juveniles where omeprazole was applied exclusively during the neurula stages (St. 14–St. 16) with juveniles where omeprazole was applied during tailbud stages (St. 18–St. 23). As previously observed, neurula stage treatment with omeprazole randomized heart position and produced some juveniles with medial hearts (7/50, two trials). In contrast, treatment with omeprazole during tailbud stages had no impact on heart positioning (50/50, right, two trials). These data suggest that ion flux specifically regulates heart asymmetry during neurula stages.

### Ion flux regulates neurula rotation

We next examined whether ion flux regulates lateral heart asymmetry through an impact on neurula rotation. During late neurula stages, *Ciona* embryos undergo a rotation around the anterior–posterior axis (Fig. 1d). To determine whether the temporal overlap between sensitivity to omeprazole treatment (see previous section) and neurula stage rotation reflects a functional link, we treated embryos with omeprazole and assayed rotation. Embryos were treated with omeprazole or DMSO just prior to neurula rotation (St. 14) and subjected to time-lapse video analysis. In control embryos, a clockwise rotation was observed. This rotation lasted until early tailbud stages and typically involved two-and-a-half turns along the axis (Additional file 2: video S1, left panel). In contrast, treated embryos show no sign of rotation (Additional file 2: video S1, right panel).

### Ion flux and chorion-dependent mechanisms synergistically regulate heart laterality

To further examine the respective contributions of ion flux and chorion-dependent mechanisms, we treated dechorionated zygotes with omeprazole. While treatment of chorionated embryos produces midline hearts in ~15% of juveniles, the combination of omeprazole treatment and dechorionation led to a dramatic increase in the penetrance of the midline heart phenotype (~52%). This increased penetrance aligns with our results on larval heart progenitor positioning in dechorionated embryos treated with omeprazole (Fig. 2c). Taken together, our results support a model in which ion flux has a dual impact on heart positioning. By influencing neurula rotation, ion flux helps to promote consistent asymmetric chorion contact, which then influences heart asymmetry. Additionally, ion flux appears to direct lateral heart asymmetry through an unknown, chorion-independent mechanism.

### Ion flux is required for polarized ciliogenesis

**Fig. 3:**
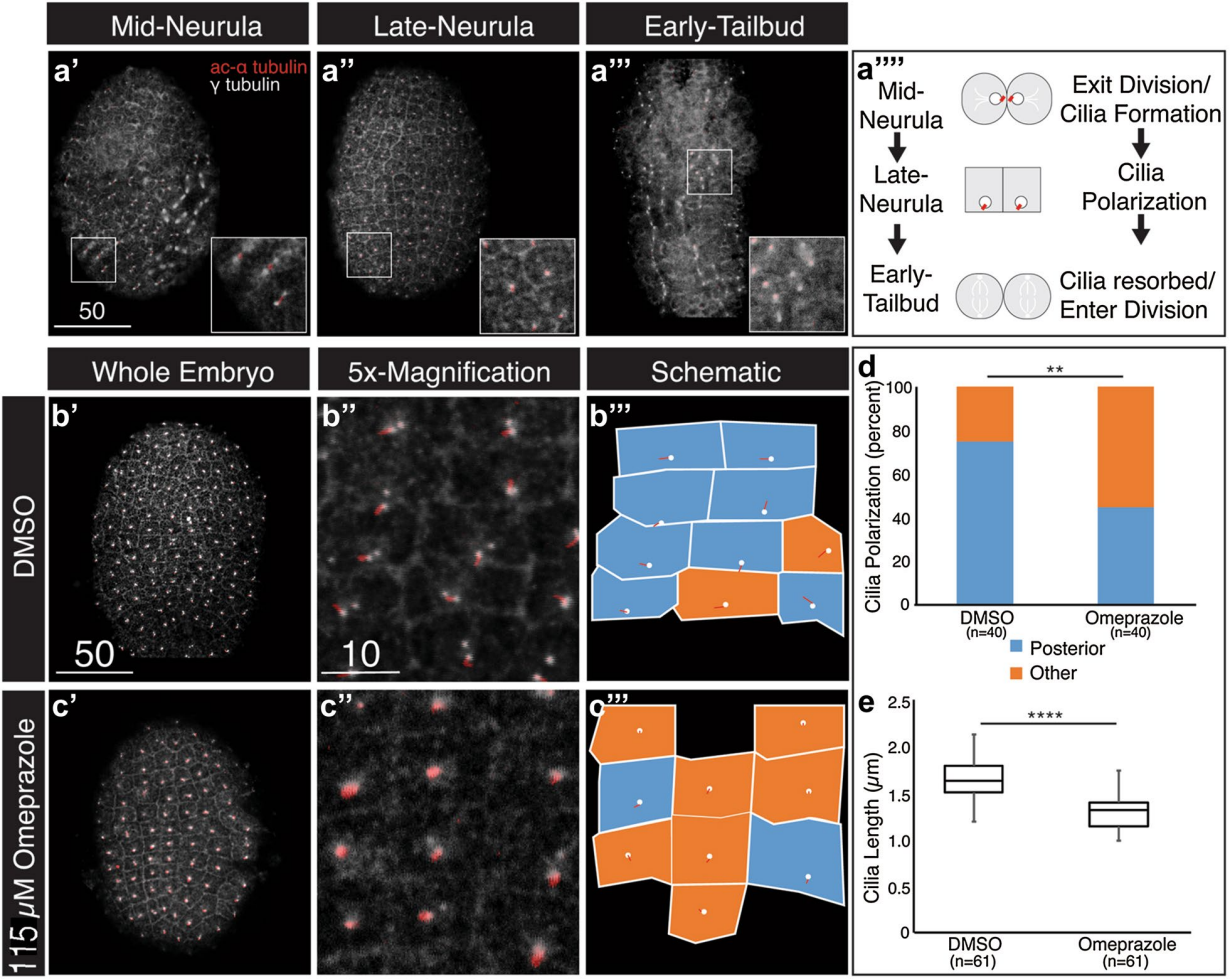
Ion flux regulates ciliogenesis in the neurula epidermis. **a**–**c** Confocal images of neurula and tailbud stage embryos stained with antibodies against acetylated alpha-tubulin (*red)* and gamma-tubulin (*white*) to demarcate ciliary microtubules and centrioles, respectively. **a′**–**a**‴ *Insets* show magnification of selected regions. **a**′′′′ Schematic showing cilia positioning during mid-neurula to early tailbud stages. **b′**–**c′** Typical cilia positioning in neurula embryos (Stage 16) treated with DMSO (**b′**) or omeprazole (**c’**) at Stage 14. **b**″–**c**″**′** Magnified views (**b**″**, c**″) and schematic illustrations (**b′**″**, c′**″) of cilia positioning. *Blue coloration* indicates posteriorly polarized cilia, and *orange coloration* indicates lack of posterior polarization (see “Methods” for details on scoring). **d** Graphical representation of data on cilia polarization in DMSO- and omeprazole-treated embryos. *Colors* correspond to (**b′**″**, c**″**′**), *n* = number of cells scored from seven embryos over two independent trials. Chi-square derived *P* value (0.006) for comparisons between DMSO versus Omeprazole-treated embryos. **e** Average cilia length in DMSO- and omeprazole-treated embryos. *Box*-and-*whisker plot* is shown. *n* represents the number of cells scored in seven embryos over two independent trials; Wilcoxon rank sum test was used to derive *P* value (3.3E−14) for comparisons between DMSO- versus omeprazole-treated embryos. In all images, anterior is *up*. *Scale bars* are in micrometers

We next began to investigate whether ion flux promotes *Ciona* neurula rotation by regulating polarized ciliogenesis. Previous studies have established that uniform cilia positioning in the vertebrate organizer is essential for generating flow [52]. Additionally, previous reports indicate that cilia are posteriorly positioned on *Ciona* epidermal cells during neurula stages [45]. Furthermore, recent work has demonstrated that ion flux is essential for ciliary growth and polarization in the *Xenopus* organizer [16]. To begin exploring whether ion flux plays a similar role in *Ciona*, we examined the morphology and localization of cilia during mid-neurula, late neurula and early tailbud stages using antibodies for acetylated alpha and gamma-tubulin (to label ciliary microtubules and centrioles, respectively, Fig. 3). Strikingly, the morphology and position of epidermal cilia appear to reflect well-characterized epidermal cell divisions during these stages (Fig. 3a′–a′′′′) [53]. At mid-neurula stage, epidermal cells are known to undergo a coordinated division [53]. As epithelial cells exit mitosis, an individual cilium forms on each daughter cell. These newly formed cilia are initially positioned as doublets spanning recently divided cell pairs (Fig. 3a′, a′′′′). A similar pattern has been observed during division in cultured mammalian cells [54]. These immature cilia are unlikely to generate a coherent flow. In late neurula stage embryos, epidermal cells do not divide. As recently formed cilia mature, they become positioned posteriorly along the cell’s apical surface and display increased length in comparison with cilia in newly divided cells (Fig. 3a″). These mature, polarized cilia may generate a flow associated with the initial embryonic rotation at this stage. Lastly, at the early tailbud stage, epidermal cells initiate an uncoordinated wave of cell divisions [53]. Epidermal cilia are resorbed in patches where cells are entering mitosis and then reform as cells exit mitosis. Resorbed cilia as well as newly formed, immature cilia are unlikely to generate flow, and this could explain why tailbud embryos no longer rotate (Fig. 3a‴). Thus, we propose that cilia-dependent flow is temporally regulated by division-dependent ciliogenesis, prompting rotation at the late neurula stage and ending rotation at the early tailbud stage (Fig. 3a′′′′).

We next determined if ion flux is required for proper epidermal ciliogenesis in neurula stage embryos. We treated embryos with either DMSO or 115 µM omeprazole beginning at the early neurula stage (St. 14). To assay for proper ciliogenesis, we fixed embryos at the late neurula stage (St. 16). At the onset of neurula rotation (St. 16), 75% of epidermal cilia in control embryos displayed typical planar polarization (Fig. 3b″, d). We also found that cilia had an average length of 1.6 µm in control embryos (Fig. 3b″, e). In embryos treated with omeprazole, cilia were often positioned close to the cell’s center and only 45% of cilia displayed posterior polarization (Fig. 3c″, d). Additionally, omeprazole treatment led to a significant decrease in cilia length (average length of 1.3 µm in treated samples vs. 1.6 µm in controls, Fig. 3c″, e). These data support a model in which ion flux polarizes newly formed cilia in epidermal cells undergoing a coordinated mitotic wave during the mid-neurula stage. According to our model, these polarized cilia then generate a flow underlying neurula rotation.

### Lack of directional protrusion in shifting heart progenitors

**Fig. 4:**
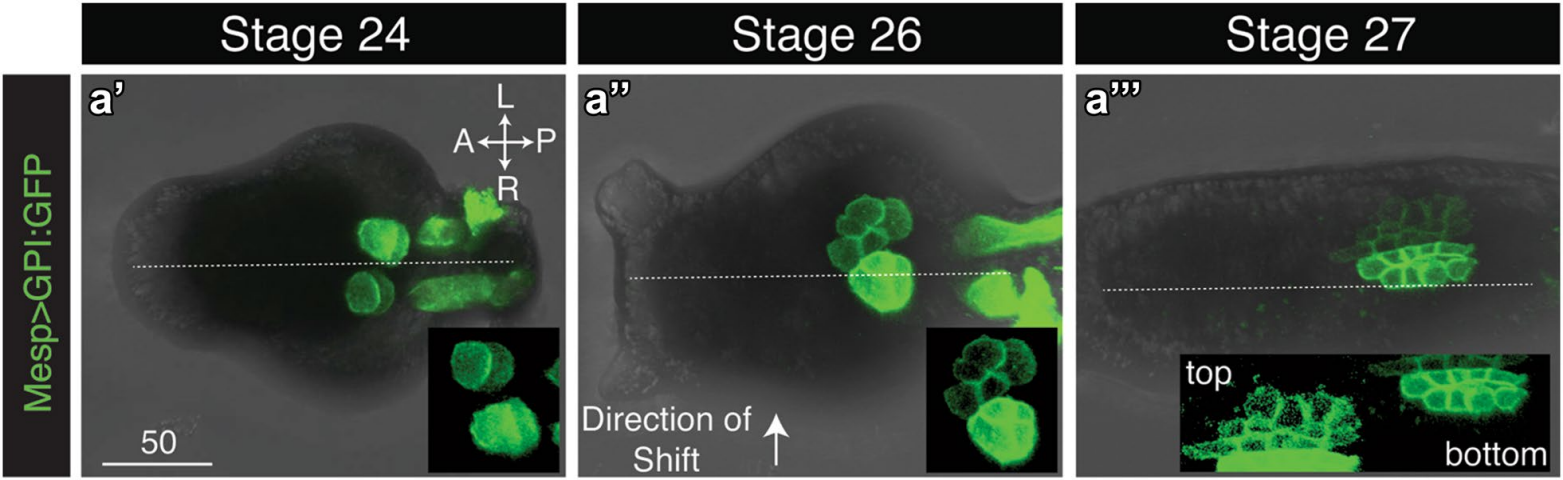
Live imaging of *Ciona* heart progenitor cells undergoing lateral shift. **a′**–**a**‴ Representative ventral projections from confocal time-lapse analysis of Mesp > GPI:GFP labeled heart progenitor membranes during Stages 24–27 (Additional file 2: video S2). Anterior to the *left*. *Scale bar* represents 50 µm

The initial asymmetric positioning of the heart progenitors (St. 25) may result from active migration or may reflect a passive shift. To distinguish between these possibilities, we performed live imaging of heart progenitor membrane dynamics using a Mesp > GPI:GFP reporter [55]. We were able to record heart progenitor position and morphology from Stages 24–27, spanning their initial shift off of the midline. Although this shift was consistently observed between Stage 24 (Fig. 4a′) and Stage 26 (Fig. 4a″), we did not observe any protrusive activity during this period (Additional file 2: video S2, Additional files 3: video S3, Additional files 4: video S4). In Stage 27 larvae, protrusive activity was sometimes observed in lateral heart progenitor cells (Fig. 4a‴). However, this protrusive activity did not consistently correspond to the direction of the lateral shift (Additional file 2: video S2, Additional files 3: video S3, Additional files 4: video S4) and may be associated with migration of the atrial siphon muscle precursors [33]. Based on these observations, we propose that asymmetric heart positioning is a passive process, perhaps driven by adhesion to neighboring tissues that are undergoing directed lateral migration or torsion.

### Lateralized endoderm morphogenesis requires ion flux

**Fig. 5:**
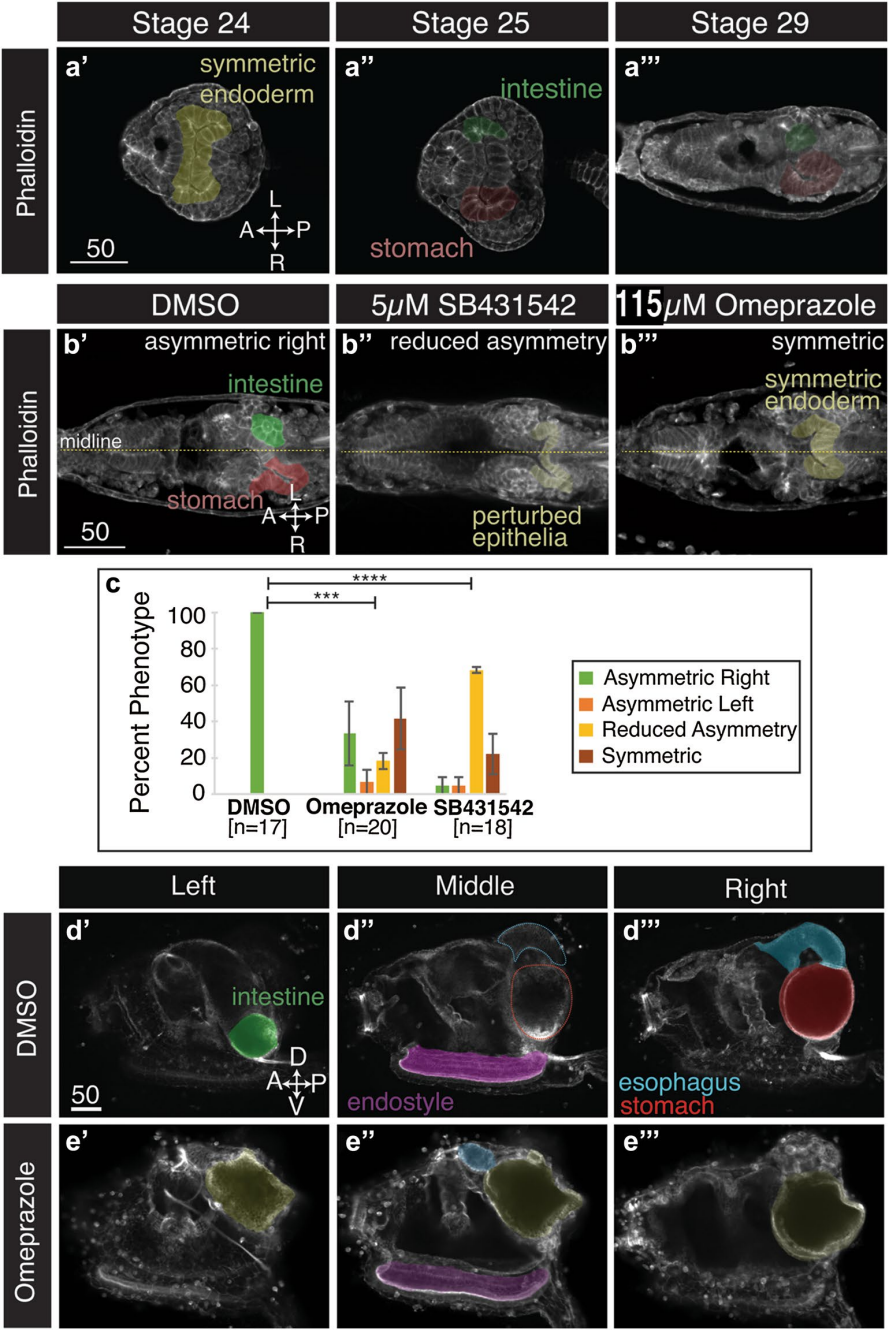
Ion flux is required for proper endoderm asymmetry. **a′**–**a**‴ Ventral sections of late embryonic and larval stages showing typical positioning of endodermal structures (*false-colored*). Cell outlines are visualized using phalloidin. **b′**–**b**‴ Ventral views of Stage 31 larvae, and endodermal structures are *false-colored*. **b′** Typical endodermal morphology in DMSO controls. **b″** Typical endodermal morphology in larvae treated with the Nodal inhibitor SB431542. **b**‴ Typical endodermal morphology in larvae treated with the ion flux inhibitor omeprazole. **c** Graphical representation of data on larval endodermal asymmetry. *Error bars* represent SEM. *n* = number of larvae scored over three-independent trials and *χ* ^2^ test derived *P* values comparing control and omeprazole-treated larva (*p* = 6.9E−4) or SB431542-treated samples (*p* = 7.6E−7). **d′**–**e**‴ Lateral sections of *Ciona* juveniles displaying endoderm asymmetry following DMSO (**d′**–**d**‴) and omeprazole neurula stage treatment (**e′**–**e**‴). Endodermal structures are *false-colored*. *Yellow* indicates symmetric endodermal organ. All *scale bars* are in micrometers

We searched for laterally asymmetric organs that could be driving the asymmetric shift observed in heart cells. Previous studies have detailed *Ciona* endoderm lateral asymmetries in larvae and juveniles, including right-sided positioning of the esophagus and stomach [56]. However, these studies have not characterized when endoderm asymmetry initially arises. To determine if *Ciona* heart asymmetry correlates temporally with endodermal asymmetry, we examined endoderm morphology in staged, phalloidin-stained samples. In these samples, endodermal rudiments are clearly delimited by columnar epithelia cells with apically enriched actin and trapezoidal hinge cells associated with folding (Fig. 5) [56]. In late tailbud II embryos (St. 24), the endoderm rudiment is roughly symmetric (Fig. 5a′). In ventral sections, the endoderm typically displays equal sized, bifurcated lateral out-pocketings. By St. 25, the ventral out-pocketing on the right bends toward the posterior while the out-pocketing on the left bends toward the anterior (Fig. 5a″). The right-sided out-pocketing is also distinguished at this stage by a more elongated, semicircular shape and appears to consist of taller, more trapezoidal shaped epithelial cells. Actin distribution on the right-sided epithelial cells was also more uniform and apically enriched. By St. 29, the right-sided ventral out-pocketing appears to elongate posteriorly (Fig. 5a‴). By contrast, the left-sided ventral out-pocketing appears to form an enclosed tube-like structure. These changes become more apparent as the larvae progress to St. 31 (Fig. 5b′). By this stage, right- and left-sided endodermal out-pocketings closely resemble previously characterized primordial stomach and intestine rudiments, respectively [56]. Thus, it appears that the initial lateral asymmetries in the heart and endoderm occur simultaneously at St. 25, supporting a model in which asymmetric endoderm morphogenesis drives heart laterality.

If endoderm morphogenesis drives heart laterality, the observed ion flux dependence and Nodal independence of heart lateral asymmetry (Fig. 2) reflect properties of endoderm morphogenesis. To test this prediction, we disrupted Nodal signaling at the neurula stage (5 µM SB431542, St. 14.5) and assayed endodermal laterality in St. 31 larvae. Larvae treated with DMSO displayed typical, asymmetric endodermal morphology (Fig. 5b′, c). In treated larvae, ventral endodermal rudiment morphology was severely disrupted and endoderm asymmetry was highly variable (Fig. 5b″, c). Additionally, epithelial cells in SB431542-treated larvae appeared to be shorter, smaller and more densely packed with atypical, basolateral actin enrichment (Fig. 5b″). Although SB431542-treated larvae sometimes displayed reduced asymmetry, it is difficult to determine whether this phenotype is due to disruption of left/right patterning or reflects general defects in endoderm morphogenesis.

We next examined the role of ion flux in lateral endoderm asymmetry. Neurula stage embryos (St. 14) treated with 115 µM omeprazole were assayed for larval and juvenile endoderm morphology. In larvae treated with omeprazole, endodermal asymmetry was often reduced or absent. In particular, ventral out-pocketings were often similar on both left and right sides (Fig. 5b‴). This result was robust and significant (~45% symmetric, 9/20; Fig. 5c). Despite the observed loss of asymmetry in treated larvae, endodermal epithelial cells displayed normal shape and apical actin enrichment (Fig. 5b″). To quantify juvenile endoderm asymmetry, we scored stomach position and found consistent right-sided stomach positioning in all DMSO-treated juveniles (30/30, right, two trials; Fig. 5d′–d‴) [56]. In omeprazole-treated juveniles, we often observed randomized stomach positioning (25/72, left; 36/72, right, two trials). In some treated juveniles, we also observed formation of a presumed esophagus along the midline anterior to a large cavity, which we categorized as a midline stomach (Fig. 5e′–e‴, 11/72, two trials). Additionally, in all juveniles scored, heart and stomach position was tightly correlated (data not shown), suggesting a causal link between lateral heart and endoderm asymmetry. Taken together, these data suggest that ion flux directly influences endoderm laterality, thereby indirectly promoting a lateral shift in the associated heart field.

## Discussion

**Fig. 6:**
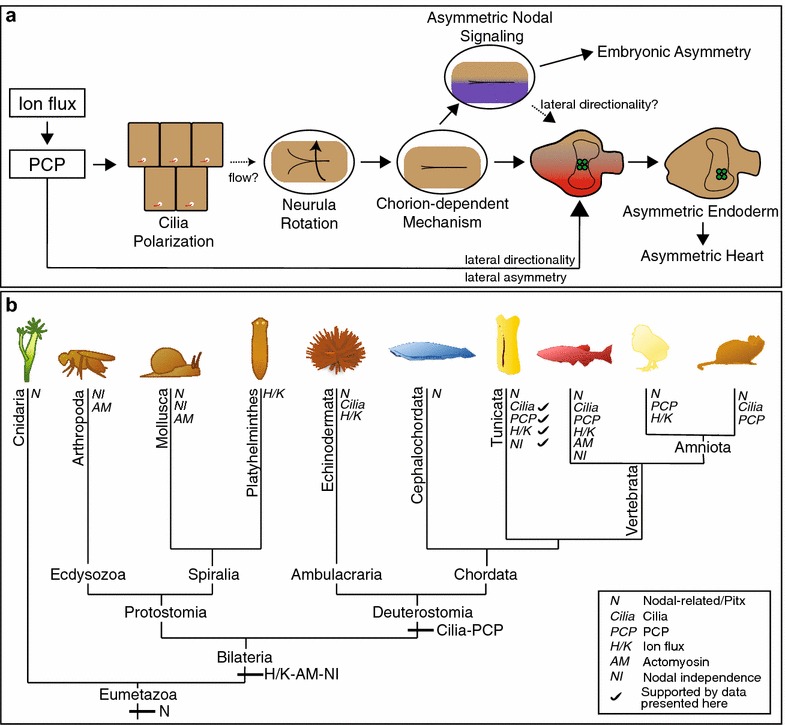
Heart asymmetry in *Ciona* and other deuterostomes. **a** Model for lateral heart and endoderm asymmetry in *Ciona*. *Solid arrows* indicated support from experimental evidence while *dotted arrows* indicated more speculative hypotheses. **b** Model for eumetazoan evolution of left/right patterning

Based on our data, we have devised a model for directional asymmetry of the *Ciona* heart and endoderm (Fig. 6a). According to our model, ion flux regulates epidermal planar cell polarity (PCP) during neurulation. PCP serves to coordinate uniform cilia positioning required for flow-dependent neurula rotation and lateral chorion contact. Chorion contact triggers asymmetric Nodal signaling and Nodal-dependent embryonic asymmetric morphogenesis. Chorion contact may also contribute to lateral heart asymmetry in a Nodal-independent manner. We hypothesize that ion flux/PCP also contributes to lateral heart asymmetry independent of both chorion contact and Nodal signaling. Further, we propose that lateralized morphogenesis of the endoderm rudiment underlies an associated lateral shift in heart progenitor position. We discuss each part of this model in the following sections.

### Ion flux-dependent PCP regulates cilia polarization

Based on our results and the relevant literature, we hypothesize that ion flux influences PCP-dependent cilia polarization in the *Ciona* neurula epidermis through a direct impact on Wnt signaling. We have shown that blocking H^+^/K^+^ ion flux disrupts cilia polarization (Fig. 3) and PCP is well established as the primary driver of cilia polarization [57–59]. The link between ion flux, PCP and cilia is best studied in relation to *Xenopus* LR patterning. In *Xenopus* embryos, ion flux is essential for proper cilia polarization in the organizer epithelium (gastrocoel roof plate) [16]. Since Wnt signaling requires an acidic extracellular environment [60, 61] and is known to regulate PCP [62], a possible link between ion flux, Wnt, PCP and cilia polarization was recently explored in *Xenopus*. It was found that knockdown of the H^+^/K^+^ ion pump (*ATP4*) disrupted both canonical and non-canonical Wnt signaling and resulted in the loss of cilia polarization. In chick, the initial symmetry breaking is cilia independent, involving leftward cell movements in Hensen’s node [14]. Directional cell movement requires ion flux and is dependent on proper PCP [14, 63]. Thus, it appears that ion flux-dependent PCP may initiate lateral asymmetry in both frog and chick. An alternative, early role for ion flux in *Xenopus* involves shifts in membrane potential along the left/right axis during early cleavage stages [15]. However, in *Ciona*, we have shown that ion flux is not required for heart lateral asymmetry prior to neurulation.

### Contributions of epidermal cilia to lateral asymmetry

We propose that polarized epidermal cilia have two distinct roles in LR patterning, serving to generate flow for neurula rotation and subsequently participating in chorion contact-mediated lateralization. Our data indicate that epidermal cilia mature and polarize precisely at the time when rotation occurs (Fig. 3), and previous studies indicate that epidermal cilia are motile at this stage [64]. Thus, epidermal cilia acquire the capability to generate flow-dependent rotation only during the neurula stage. Intriguingly, our data indicate that the timing of cilia-dependent rotation is dictated by epidermal cell division patterns (Fig. 3). Following rotation, it is possible that cilia participate in chemosensory or mechanosensory functions associated with chorion contact. This dual role would mirror the established two-cilia model for left/right patterning in vertebrate embryos. In mice, frogs and fish, motile organizer cilia generate a directional flow that triggers non-motile cilia in an asymmetric manner, resulting in the LR asymmetric signaling cascade [5–7, 65–69].

### Nodal-independent contributions to lateral asymmetry

The relative contributions of ion flux, chorion contact and Nodal signaling to LR patterning remain difficult to untangle. We found that inhibition of ion flux disrupted both lateral directionality and asymmetry while dechorionation and pharmacological inhibition of Nodal had no impact on lateral asymmetry (Fig. 2). These results suggest that ion flux contributes to a LR asymmetry pathway that is independent of both Nodal and chorion contact. However, we also found that dechorionation enhances the impact of ion flux inhibition on lateral asymmetry, suggesting overlapping roles for ion flux and chorion contact. Nodal-independent lateral asymmetries in vertebrate embryos appear to involve polarized cytoskeletal dynamics. In zebrafish, Nodal-independent cardiac dextral looping is regulated by tissue intrinsic polarization of myosin [27]. In chick, the initial lateral asymmetries in the morphogenesis of Hensen’s node are also Nodal independent and appear to involve planar cell polarization of cytoskeletal dynamics [63]. Thus, we are interested in investigating whether Nodal-independent lateral asymmetries in *Ciona* heart and gut morphogenesis also involve polarized cytoskeletal dynamics. We are also interested in investigating how the initial ion flux-dependent/Nodal-independent asymmetries generated in neurula stage embryos are translated into subsequent lateralization of heart and gut morphogenesis.

### Relationship between lateral asymmetries in endoderm and heart morphogenesis

We propose that ion flux-dependent lateralization of endoderm morphogenesis underlies a passive, rightward shift in heart field position. According to this model, heart progenitors are tightly adherent to the adjacent endoderm. Thus, active rightward out-pocketing of the endoderm (Fig. 5) leads to passive rightward displacement of the heart progenitors. In support of this model, heart progenitors display no directed protrusive activity while they are initially shifting off of the midline (Fig. 4). Further, the initial asymmetries in heart and endoderm rudiments arise simultaneously at St. 25 in an ion flux-dependent manner (Figs. 2, 4). Additionally, in St. 21 late tailbud stage embryos, prior to their lateral shift at St. 25 larvae, heart progenitors appear to be embedded in the endoderm rudiment [31] and this close physical proximity is maintained in later stages. Thus, continued adhesion to endoderm epithelial cells or associated matrix in larval stages may facilitate heart progenitor displacement.

### Evolution of LR patterning

Based on our data and the published literature, we have drawn out an evolutionary model regarding ancestral modes of LR patterning within the eumetazoans (Fig. 6b). A Nodal-related/Pitx module is generally considered to play an ancestral role in eumetazoan LR patterning. However, accumulating evidence suggests that a Nodal-independent pathway, involving ion flux or tissue intrinsic cytoskeletal polarity, may also play a deeply conserved role in lateral asymmetry among the bilaterians [1, 4]. Inhibitor studies indicate that ion flux contributes to LR patterning in both vertebrates and non-chordate deuterostomes, including tunicates and echinoderms [17, 70]. Additionally, there is some evidence that ion flux contributes to LR patterning in planarians [71]. As discussed earlier, zebrafish heart formation and the initial asymmetric morphogenesis of the chick node appear to involve Nodal-independent cytoskeletal polarization [14, 27]. In protostome molluscs, polarized cytoskeletal elements function upstream of laterally asymmetric Nodal signaling [72, 73]. In the ecdysozoans, *Drosophila* and *C. elegans*, Nodal genes are absent [74] and lateral asymmetries are solely based on cell intrinsic cytoskeletal polarity [75, 76]. Widespread molecular similarities in ion flux or cytoskeletal pathways contributing to lateral asymmetry among bilaterians would strengthen the case for ancestral acquisition of this pathway.

We further propose that cilia-dependent LR patterning may have arisen within the ancestral deuterostome lineage. A central role for cilia in mouse, zebrafish and frog LR patterning is well established [5, 7, 67]. Accumulating data from this and other studies are beginning to clarify the role of cilia in tunicate LR patterning. Recently, sea urchins have also been found to deploy cilia for LR patterning [77, 78]. The involvement of cilia in LR patterning in a wide range of deuterostomes suggests that this represents an ancestral mechanism that was lost in some vertebrates, including chick and pig embryos. To further test this proposed evolutionary model, future studies should focus on testing whether cilia influence LR patterning in amphioxus and hemichordates. Additionally, further characterization of the upstream and downstream factors linking cilia to other LR patterning components and whether these components are broadly conserved within the deuterostomes will be critical in delineating how evolutionary shifts in LR patterning contributed to diversification in this clade.

## Additional files

**Additional file 1: Figure S1.** Impact of SB431542 treatment on *pitx* expression. In situ results showing typical *pitx* expression in the heads of tailbud stage embryos (St. 22) dechorionated shortly before fixation and treated as indicated. **(a’)** Wild-type expression pattern. **(a”)** 5-μM treatment leads to loss of lateral *pitx* expression in the epidermis and internal germ layers, and note that Nodal-independent expression in the anterior neural boundary (Yoshida and Saiga, 2008) is not impacted. **(a”’)** 0.05-μM treatment appears to reduce *pitx* expression, particularly in the left epidermis, but does not eliminate Nodal-dependent lateral expression. The *Ci-pitx* probe was generated using T7 polymerase on the *Ci-pitx* cDNA in Gene Collection library clone GC30b01 (Satou et al., 2002) according to standard protocols. To ensure that dechorionation did not interfere with left-right patterning, embryos were dechorionated just prior to fixation using the same protocol as employed for zygotic dechorionation. Standard in-situ hybridization protocols were also employed (Cooley et al., 2011).

**Additional file 2: video S1.** Ion flux is required for neurula rotation. Time-lapse of DMSO-treated (left panel) and 40-μM omeprazole-treated (right panel) neurula stage embryos. Rotation is apparent on the left, while rotation is absent on the right. Movement at the end of the video in the right panel is associated with tail morphogenesis.

**Additional file 3: video S2.** Live imaging of *Ciona* heart progenitor cells. Ventral projections from one of three confocal time-lapse videos of three different embryos expressing Mesp>GPI::GFP to label heart progenitors. No consistent protrusive activity is observed. Protrusive activity after heart progenitors have shifted is associated with ASMPs.

**Additional file 4: video S3.** Live imaging of *Ciona* heart progenitor cells. Ventral projections from one of three confocal time-lapse videos of three different embryos expressing Mesp>GPI::GFP to label heart progenitors. No consistent protrusive activity is observed. Protrusive activity after heart progenitors have shifted is associated with ASMPs.

**Additional file 5: video S4.** Live imaging of *Ciona* heart progenitor cells. Ventral projections from one of three confocal time-lapse videos of three different embryos expressing Mesp>GPI::GFP to label heart progenitors. No consistent protrusive activity is observed. Protrusive activity after heart progenitors have shifted is associated with ASMPs.

## Abbreviations

LR: left/right
FGF: fibroblast growth factor
HPF: hours post-fertilization
PCP: planar cell polarity
