# Supplementary material for: Establishment of lateral organ asymmetries in the invertebrate chordate, Ciona intestinalis
Source: EvoDevo. 2017 Jul 25;8:12. doi: 10.1186/s13227-017-0075-9 (PMC5526266; doi:10.1186/s13227-017-0075-9)
Supplement: Supplementary file 1 — Additional file 1: Figure S1. Impact of SB431542 treatment on pitx expression. In situ results showing typical pitx expression in the heads of tailbud stage embryos (St. 22) dechorionated shortly before fixation and treated as indicated. (a’) Wild-type expression pattern. (a”) 5-μM treatment leads to loss of lateral pitx expression in the epidermis and internal germ layers, and note that Nodal-independent expression in the anterior neural boundary (Yoshida and Saiga, 2008) is not impacted. (a”’) 0.05-μM treatment appears to reduce pitx expression, particularly in the left epidermis, but does not eliminate Nodal-dependent lateral expression. The Ci-pitx probe was generated using T7 polymerase on the Ci-pitx cDNA in Gene Collection library clone GC30b01 (Satou et al., 2002) according to standard protocols. To ensure that dechorionation did not interfere with left-right patterning, embryos were dechorionated just prior to fixation using the same protocol as employed for zygotic dechorionation. Standard in-situ hybridization protocols were also employed (Cooley et al., 2011). [file 13227_2017_75_MOESM1_ESM.pdf]

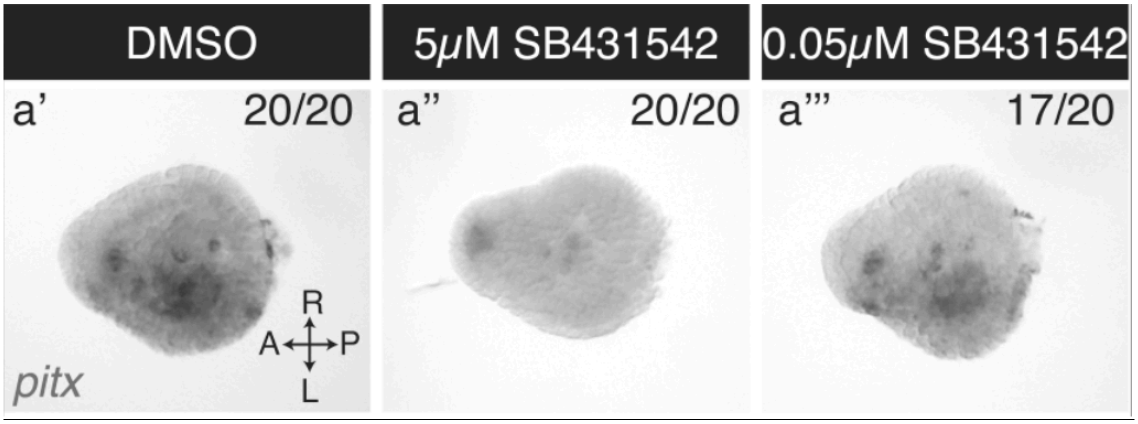

Figure S1. Impact of SB431542 treatment on *pitx* expression. In-situ results showing typical *pitx* expression in the heads of tailbud stage embryos (St. 22) dechorionated shortly before fixation and treated as indicated. **(a')** Wildtype expression pattern. **(a'')** 5μM treatment leads to loss of lateral *pitx* expression in the epidermis and internal germ layers, note that Nodal-independent expression in the anterior neural boundary (Yoshida and Saiga, 2008) is not impacted. **(a''')** 0.05μM treatment appears to reduce pitx expression particularly in the left epidermis, but does not eliminate nodal-dependent lateral expression. The *Ci-Pitx* probe was generated using T7 polymerase on the *Ci-Pitx* cDNA in Gene Collection library clone GC30b01 (Satou et al., 2002) according to standard protocols. To ensure that dechorionation did not interfere with left-right patterning, embryos were dechorionated just prior to fixation using the same protocol as employed for zygotic dechorionation. Standard in-situ hybridization protocols were also employed (Cooley et al., 2011).

To view supplemental videos, please use the following link:

https://drive.google.com/drive/folders/0B39IHb5TppUKZ2duVjc5ZklXQzA?usp=sharing

Video S1. Ion flux is required for neurula rotation. Time-lapse of DMSO-treated (left panel) and 40μM omeprazole-treated (right panel) neurula stage embryos. Rotation is apparent on the left, while rotation is absent on the right. Movement at the end of the video in the right panel is associated with tail morphogenesis.

Videos S2-4. Live-imaging of *Ciona* heart progenitor cells. Ventral projections from a confocal time-lapse of 3 different embryos expressing Mesp>GPI::GFP to label heart progenitors. No consistent protrusive activity is observed. Protrusive activity after heart progenitors have shifted is associated with ASMPs.
